# Motivation as a function of success frequency

**DOI:** 10.1007/s11031-021-09904-3

**Published:** 2021-09-30

**Authors:** Katinka van der Kooij, Lars in ‘t Veld, Thomas Hennink

**Affiliations:** Department of Human Movement Sciences, Vrije Universiteit Amsterdam, Van der Boechorststraat 9-1, 1081BT Amsterdam, The Netherlands

**Keywords:** Motivation, Reward, Success, Intrinsic motivation, Flow

## Abstract

It is well-established that intermediate challenge is optimally motivating. We tested whether this can be quantified into an inverted-U relationship between motivation and success frequency. Participants played a game in which they navigated a scene to catch targets. In Experiment 1 (*N* = 101), play duration was free and the motivating value of success frequency was measured from the probability that a player would continue at that frequency. In Experiment 2 (*N* = 70), play duration was fixed, and motivation was measured using repeated self-reports. In Experiment 1, the probability to continue increased linearly with the success frequency whereas play duration did show the inverted-U relationship with success frequency. In Experiment 2, self-reported motivation showed the inverted-U relationship with success frequency. Together, this shows that motivation depends on success frequency. In addition, we provide tentative evidence that the concept of intermediate challenge being most motivating can be quantified into an inverted-U relationship between motivation and success frequency.

## Introduction

The tasks that motivate you are probably tasks that you are skilled at. All contemporary theories about motivation include positive effects of a concept related to beliefs about competence (Cook & Artino, 2016). As beliefs about competence enhance motivation, it seems evident that success experiences enhance motivation as well because they provide information about success (Kukla, 1972). However, the positive relationship between competence and motivation does not imply that constant success is optimal. Failing occasionally might be motivating because it enhances the value of success (Atkinson, 1957; Kukla, 1972), evokes arousal (Nygard, 1975), or because it signals that challenge and skill are in balance (Csikszentmihalyi, 1990; Steels, 2004). Hence a success frequency lower than 1 might be optimal. In this study, we assess how motivation for an online computer game depends on the success frequency. In doing so, we define motivation as the tendency to engage with a task.

The theory of achievement motivation (McClelland et al., 1953) is best known for defining two complementary motives of which the composition differs between individuals: a motive to succeed and a motive to avoid failure. The theory also describes motivation as a multiplicative function of three factors: the motive (succeed, avoid failure), the probability of success, and the value of success. The value of success decreases with the probability of success, and therefore the apex of motivation is predicted where the value and probability function cross: at a success probability of 0.5. Indeed, in a study that took performance as a measure of motivation, performance on a math task was higher when students were informed that the success probability in their experimental group was 0.3 or 0.5 compared to when they were informed that the success probability was 0.05 or 0.75 (Atkinson, 1957). However, the relationship between motivation and the probability of success depends on several factors. First, for individuals with a strong failure avoidance motive, motivation peaks at a success probability of 1 where failure is absent or at a success frequency of 0 where failure is least costly. Second, the relationship has been found most poignant when individuals attribute the success to the effort invested in the task (Kukla, 1972). And finally, arousal might mediate the relationship between motivation and the probability of success (Nygard, 1975). A task needs to require effort for it to arouse motivation and hence the task needs to be difficult to be motivating (Brehm & Self, 1989).

Flow Theory (Csikszentmihalyi, 1990) takes a more qualitative approach and focuses on flow, a mental state encompassing both focused attention and motivation. Flow depends on a balance between perceived challenge and perceived skill. Too little challenge results in boredom, whereas too much challenge results in anxiety. This idea is well supported by experimental evidence. For instance, student’s self-reports of flow were largest when differences between self-reported challenge and skill were minimal (Moneta & Csikszentmihalyi, 1996). Also, a game whose difficulty was constantly matched to the participant's performance induced more flow compared to the same game set at a constant low or high difficulty (Keller et al., 2011). As another example, flow experiences while playing off-the-shelf computer games were affected by the combination of experienced challenge and skill (Jin, 2012). And, building on the assumption that motivation is associated with greater neural responses, near-defeats and narrow-wins in a computer game resulted in stronger neural responses than either complete-defeats or easy-wins (Ma et al., 2017a).

Flow theory doesn’t include concepts of success and failure but the balance between challenge and skill could be formulated in terms of a success frequency. On the one hand, one might expect challenge and skill to be in balance when one is always successful, and the success frequency is 1. However, when would the challenge be too low then? Constant success might signal too little challenge. Challenge and skill might be in balance when a task can be learned rather than when it is mastered. Success frequencies that exceed chance level but are lower than 1 might signal this. Hence, Flow Theory can be interpreted as supporting the idea that the relationship between motivation and the success frequency follows an inverted U.

The proposed inverted-U relationship between motivation and the probability of success, a concept closely related to the success frequency, has been tested by manipulating the perceived success probability with instructions (Atkinson, 1957). If we could define motivation as a function of an observable property of the task, this would facilitate the application of motivation theories in the design of digital tasks. One such property could be the success frequency defined as the fraction of successes over a recent history. In an earlier study on motivation for a virtual reality ‘fly catching’ task, we manipulated the success frequency to be 0.3, 0.5, or 0.7. The results showed that motivation depended positively on the success probability (van der Kooij et al., 2018). There was no evidence of an inverted-U relation. However, we cannot exclude the possibility that motivation decreases for success frequencies higher than 0.7. Another study among older adults showed that motivation for a sequence learning game was highest when the progression to a new sequence was tied to the success frequency exceeding 0.7 compared to when the sequences were presented in random order or in a blocked order (Beik & Fazeli, 2021). Thus, motivation might depend on the success frequency following an inverted U with a peak around an intermediate success frequency of 0.7.

In the current study, we test the hypothesis that there is an inverted-U relationship between motivation and success frequency. We test this hypothesis in two experiments using an online browser game as the experimental task. In Experiment 1, we focus on the motivation to engage with the task and measure the probability that a participant will continue for a certain success frequency. In Experiment 2, we assess experienced motivation in a constrained setting (Atkinson, 1957) where the participant has to engage with the game for thirty minutes, and assess how the intensity of experienced motivation varies as a function of the experienced success frequency using self-reports of motivation (van der Kooij et al., 2019).

## Experiment 1

### Design

We tested the relationship between motivation and the success frequency in a non-experimental design in which all participants performed the same task.

### Participants

In Experiment 1, 101 participants (50 male, 51 females; mean age 25 ± 8 years) took part. They were recruited through the personal network of the students who ran the study, through the VU University social media channels, and through the university’s online system for study participation. Five participants were excluded because they played fewer than ten trials. No a-priori power analysis was performed. We had no prior information on the range of success frequencies that would be measured in the task and therefore we had no estimate of the variability in the outcome parameter.

### Task

**Fig. 1:**
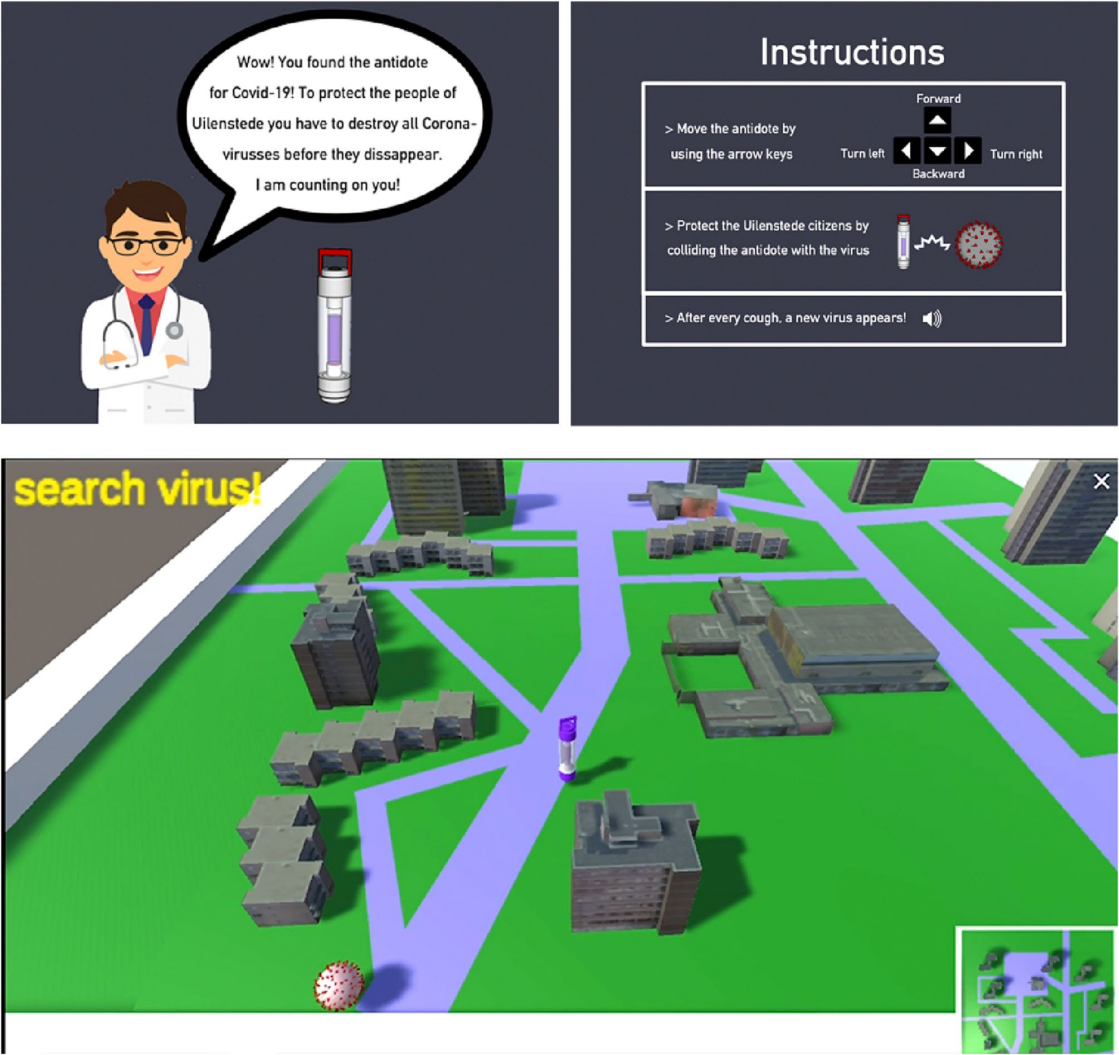
Methods. (Top panels) Instructions. (Bottom panel) Screenshot of online game with the virus, antidote avatar, and, buildings around which the player had to navigate

Participants played an online game in which their task was to catch viruses with an ‘antidote’ avatar that could be moved with the arrows on the keyboard. They were first explained the game narrative of protecting the university campus by destroying corona viruses. Participants were instructed that they had to catch the viruses before they disappeared by moving an antidote avatar with the arrow keys on the keyboard (Fig. 1A). They were instructed to play as long as they wanted and to close the browser when they wanted to quit.

The scene where viruses appeared depicted a field with buildings which served as obstacles that could occlude the position of the virus (Fig. 1B). Viruses were initiated at a random location in the scene for a limited lifetime. For the first 10 trials, the lifetime was 7 s. After that the lifetime was updated every ten trials randomly chosen between 5, 7, or 9 s. A trial started with a cough sound and the appearance of a new virus. The player had to catch the virus by navigating towards it, avoiding the buildings in the scene. The virus remained on the scene until it was caught, or the end of its lifetime was reached. Whether the virus was caught was additionally communicated by a 'virus caught' or 'virus missed' text displayed for 500 ms on the top left of the screen. After that, a new trial started with the appearance of a virus at a random location in the scene.

### Procedure

Participants visited a website where they read the study information and where they provided informed consent. Participants were informed that they would participate in a study on motivation but received no further information on the purposes of the experiment. They were informed that they would play an online game in which their task was to catch viruses by moving an avatar with the arrows on the keyboard. After providing informed consent, they accessed the online game where they entered their gender and age.

### Measures and data analysis

**Fig. 2:**
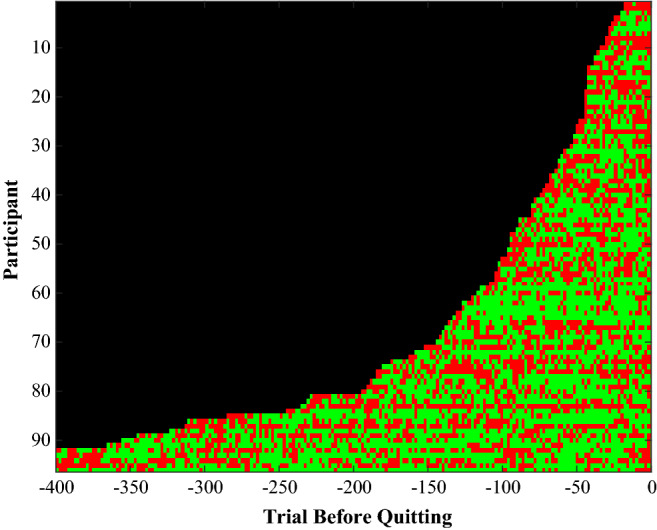
Success and failures Experiment 1. The pattern of successes (green) and failures (red) experienced by the 96 participants in the study (Color figure online)

To ensure that we only used trials in which the participant was engaged in the task we excluded trials in which the participant made no keypresses. This resulted in the exclusion of 0.02% of the trials. The independent variable was the success frequency, which was the mean success (which could take a value of 0 or 1) over a history of ten trials. The history of ten trials was based on pilot data showing that participants already started to quit after a small number of trials (see Fig. 2). For the first nine trials, we averaged over the available trials.

The primary outcome parameter was the probability to continue, which we denote as ‘pContinue’. To calculate this value, we binned the success frequencies on the different trials in nine bins between 0.1 and 1. These bins contained trials after which the participant quit and trials after which the participant continued. To obtain the pContinue, we counted the number of trials after which the participant continued and divided this value by the total trial count in the bin. As an example, if all participants would continue when encountering a success frequency between 0.9 and 1, this would result in pContinue of 1 for this bin. In contrast, if all participants would quit, the pContinue would be zero. To cross-check the results obtained with our primary measure, we obtained one secondary parameter of motivation: the play duration in minutes.

To test for an inverted-U relationship between motivation and the success frequency we performed a quadratic regression as in (Ma et al., 2017a, 2017b). To test whether this model described the data better than a linear model, we also performed a linear regression on the motivation (M) as a function of success frequency (sF), using the build-in Matlab function ‘fitlme’. To accommodate for the fact that some frequencies might be rarer than others, we weighted the data by the number of trials on which the success frequency occurred. The following equations were used:

$${\text{Linear}}:M\sim b1 + b2 \times sF$$

$${\text{Quadratic}}:M\sim b1 + b2 \times sF^{2} + b3 \times sF$$

We first assessed the relation between success frequency and motivation on a trial-by-trial basis by regressing the pContinue onto the success frequency. As a secondary analysis, we assessed how the play duration depended on the mean success frequency in the task using the same regressions. The model fits were compared with a likelihood ratio test using the build-in Matlab function ‘compare.’ MatLab code used for data processing and analysis can be downloaded from the Open Science Foundation: https://osf.io/ag86v/.

## Results

The number of trials played varied between 10 and 359 (Fig. 2). Of the trials, 0.02% were excluded because the player was inactive on that trial.

**Fig. 3:**
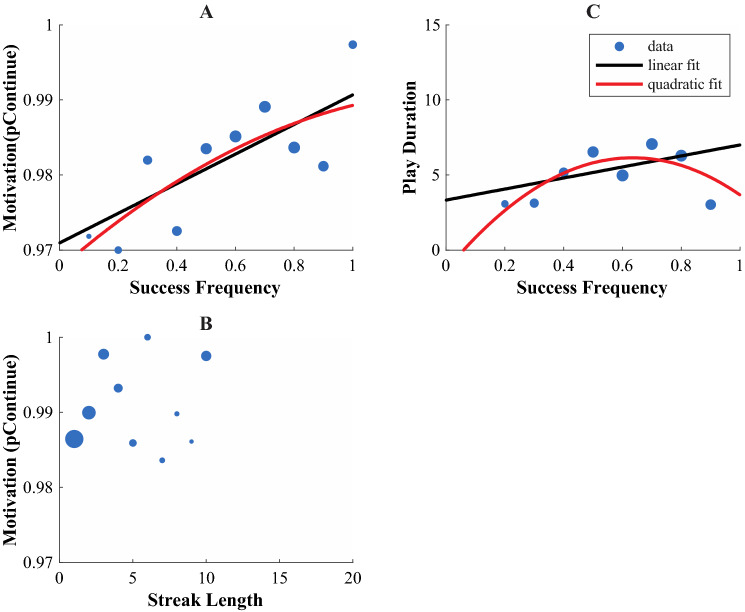
Motivation and success frequency Experiment 1. **A** The probability to continue (pContinue) as a function of success frequency, with linear and quadratic fit. **B** Play duration as a function of success frequency, with linear and quadratic fit. **C** pContinue as a function of streak length. Dot size represents the number of trials the data point was based on

The median number of trials was 48. Hence, the median participant continued after 47 trials and quit after 1 trial. Therefore, the ‘baseline’ pContinue was 47/48 = 0.98. Figure 3A shows the probability to continue as a function of the success frequency.

The results of the regression are shown in Table 1. The linear regression showed that the probability to continue depended positively on the success frequency (*b*2 = 0.02, *SE* = *0.01*, t = 3.13, *p* = 0.03). The quadratic regression showed no relation between the probability to continue and the success frequency (*b*2 = 0.03, *SE* = 0.03, *t* = 1.15, *p* = 0.29; *b*3 = − 0.01, *SE* = 0.02, *t* = − 0.49, *p* = 0.64). In contrast to our prediction, the likelihood ratios test showed that the quadratic fit did not describe the data better than the linear fit [*LR*(1) = 0.24, *p* = 0.62]. Figure 3A shows that especially the probability to continue at a success frequency of 1 deviated from the inverted-U relationship. This might have been found because this success frequency implied a streak of ten successes in a row whereas the other success frequencies did not imply a streak. Therefore, we plotted the probability to continue as a function of streak length. Figure 3B shows that the probability to continue increased with streak length.

**Table 1:** Regression analysis examining the effects of success frequency and block number on the motivation

Probability to continue						
	Linear regression			Quadratic regression		
Fixed effects	*B*	*SE* (*B*)	*t*	*B*	*SE* (*B*)	*t*
Intercept (*b*1)	0.97	0.01	235.24***	0.97	0.01	120.1***
Success Frequency (*b*2)	0.02	0.01	3.13*	0.03	0.03	1.15
Success Frequency Squared (*b*3)				− 0.01	0.02	− 0.49

Play duration						
	Linear regression			Quadratic regression		
Fixed Effects	*B*	*SE* (*B*)	*t*	*B*	*SE* (*B*)	*t*
Intercept (*b*1)	3.33	1.27	2.62*	− 1.34	2.25	− 0.59
Success Frequency (*b*2)	3.67	2.16	1.7	23.59	8.8	2.68*
Success Frequency Squared (*b*3)				− 18.59	8.06	− 2.31

^*^p < 0.05

^**^p < 0.01

^***^p < 0.001

The secondary outcome parameter, the play duration, was left-skewed. Therefore, we calculated the median play duration for each bin of success frequency instead of the mean play duration. The linear regression (Fig. 3C) showed no effect of success frequency on play duration (*b*2 = 3.67, *SE* = 2.16, *t* = 1.7, *p* = 0.14). The quadratic regression did show an effect of success frequency on play duration (*b*2 = 23.59, *SE* = 8.79, *t* = 2.68, *p* = 0.04; *b*3 = − 18.59, *SE* = 8.06, *t* = − 2.31, *p* = 0.07). The likelihood ratios test showed that the quadratic fit described the data marginally better than the linear fit [*LR*(1) = 4.08, *p* = 0.04]. The apex of this fit falls at a frequency of 0.63.

In a final set of analyses, we considered the characteristics of the task. To introduce variations in the success frequency, the lifetime of the virus was varied. Variations in the lifetime indeed introduced variations in the success frequency (0.43 ± 0.49 for a lifetime of 5 s, 0.56 ± 0.5 for a lifetime of 7 s, and 0.74 ± 0.44 for a lifetime of 9 s). In contrast, the number of participants that quit during a block with one of the three lifetimes did not depend much on the lifetime (31 for a lifetime of 5 s, 36 for a lifetime of 7 s and 29 for a lifetime of 9 s).

## Discussion experiment 1

In Experiment 1, we tested for an inverted-U relationship between motivation and success frequency. In contrast to the prediction, the probability to continue increased linearly with the success frequency. This seems in conflict with the idea that there is an inverted-U relationship between motivation and the probability of success (Atkinson, 1957). The results are consistent however with our previous finding that self-reported motivation in a virtual reality task depends positively on the success frequency (van der Kooij et al., 2018). For the play duration, the quadratic model described the data marginally better with a predicted peak motivation at a success frequency of 0.63, a slightly higher frequency than the peak motivation frequency proposed by theory of achievement motivation (Atkinson, 1957).

The main strength of Experiment 1 was that we measured motivation from the participants' engagement in a task. Using the probability to continue as an outcome parameter also caused statistical limitations because the model fits were based on only ten data points. Moreover, the data were collapsed across participants. Therefore, we could not account for inter-participant variability in our data analysis. Finally, the inverted-U relationship might have been obscured by a frequency of 1 implying a streak of ten successes which the participant might not want to quit.

## Experiment 2

In Experiment 2, we again tested the hypothesis that there is an inverted-U relationship between motivation and the success frequency but this time we used a different design in which we collected repeated self-reports of motivation for each participant. A fixed play duration was used to in order to measure motivation independent of breaking a streak.

## Methods

### Participants

In Experiment 2, 70 psychology students (7 male, 63 females; mean age 20 ± 1.7 years) took part. They were recruited through the university's online system for study participation and received course credits for their participation. Six participants were excluded because they did not complete the experiment. Based on pilot studies we estimated to need at least 60 participants.

### Procedure

To measure motivation, we used a Quick Motivation Index (QMI, van der Kooij et al., 2019) in which participants rated their enjoyment and motivation to continue on a scale of 1 to 7 following each block of ten trials. Participants responded to the following two questions using a slider, initiated at a value of 1 (not at all) and with a maximum of 7 (very much):

- *How much do you enjoy the game until now?*
- *How motivated are you to continue?*

The game was identical to the game used in Experiment 1 besides three changes. First, unlike Experiment 1, we initialized the virus lifetime for the first 10 trials randomly choosing between 5, 7, and 9 s. Second, the play duration was fixed to 15 blocks of 10 trials. In total, the experiment lasted about 30 min. Third, a progress bar represented the trial number.

### Measures and data analysis

Our primary independent variable was the success frequency, which was calculated as the mean success in a block of ten trials. The main outcome parameter was the mean score on the two QMI items. This measure has not been validated but we previously found (van der Kooij et al., 2019) that the measure correlates well with the Intrinsic Motivation Inventory (Ryan, 1982) which has adequate reliability (McAuley & Duncan, 1989). In addition, experience sampling methods have demonstrated the value of using repeated administration of single-item measures (Moneta & Csikszentmihalyi, 1996).

To test whether there is an inverted-U relationship between motivation and the success frequency, we compared the fit of a quadratic and a linear model. For model fitting, we used multi-level regression analysis on the motivation (*M*) in which the success frequency (*sF*) and block were predictors, and the participant was a random factor:

$$\normalsize \it {\text{Linear}}:M\sim b1 + b2 \times {\text{block}} + b3 \times sF + \left( {{1}|{\text{participant}}} \right)$$

$$Quadratic:M \sim b1 + b2 \times block + b3 \times sF + b4 \times sF^{2} + (1|participant)$$

Model fits were compared with a likelihood ratio test using the build-in MatLab function ‘compare’.

## Results experiment 2

**Fig. 4:**
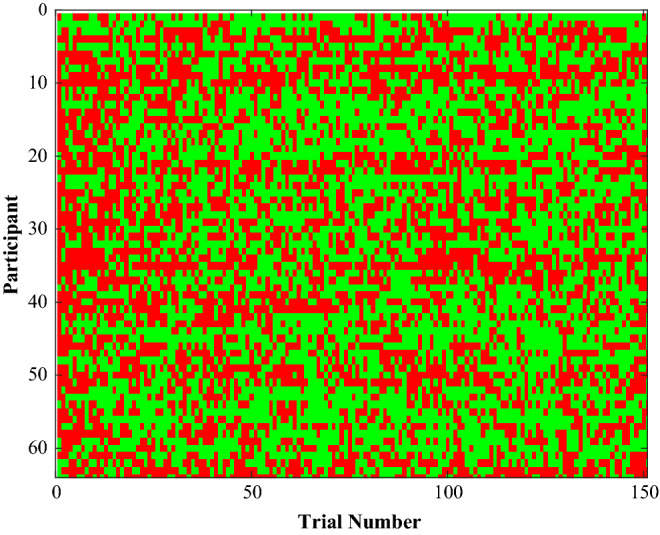
Successes and failures Experiment 2. The pattern of successes (green) and failures (red) experienced by the 66 participants in the study (Color figure online)

**Fig. 5:**
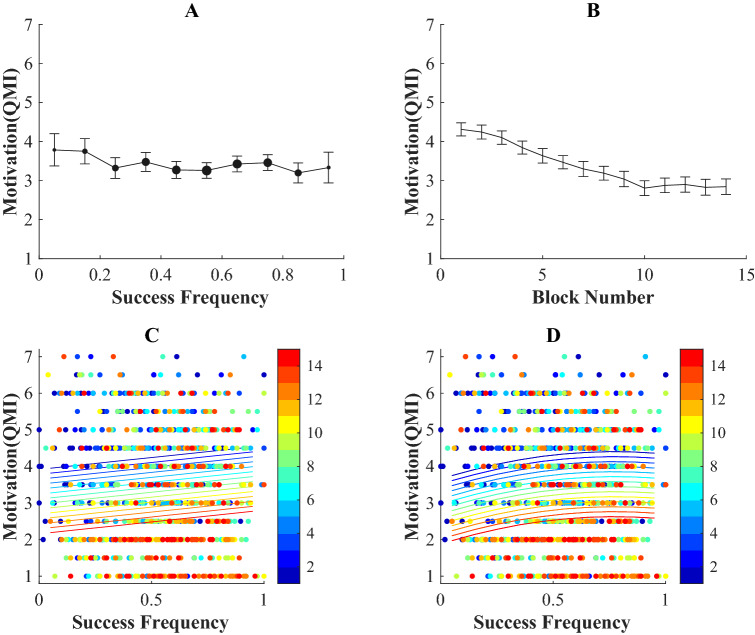
**A** Motivation and success frequency Experiment 2. **A** Mean QMI score within ten success frequency bins. Dot size indicates the number of trials in the bin and the error bar denotes the SE. **B** Mean QMI score with SE as a function of block number. **C** and **D** QMI score as a function of success frequency with the different blocks colour coded. **C** Data and linear fit. **D** Data and quadratic fit

As in Experiment 1, the task resulted in a pattern of successes and failures (Fig. 4A). We tested how self-reported motivation depended on the success frequency (Fig. 5A) and the block number (Fig. 5B) by performing a linear (Fig. 5C) and a quadratic regression (Fig. 5C).

Results of the regression analysis are shown in Table 2. In the linear model, both terms were significant. There was a negative effect of block number, *b*2 = − 0.14, *SE* = 0.01, *t* = − 17.6, *p* < 0.01 and a positive effect of success frequency, *b*3 = 0.65, *SE* = 0.18, *t* = 3.66, *p* < 0.01. In the quadratic model also all terms were significant. There was a significant negative effect of block number, *b*2 = − 0.14, *SE* = 0.1, *t* = − 17.76, *p* < 0.01. In addition, there was a significant effect for the success frequency, *b*3 = 1.94, *SE* = 0.63, *t* = 3.08, *p* < 0.01, and the squared success frequency, *b*4 = − 1.25, *SE* = 0.59, *t* = − 2.14, *p* = 0.03.

**Table 2:** Regression analysis examining the effects of success frequency and block number on the motivation

Probability to continue						
	Linear regression			Quadratic regression		
Fixed effects	*B*	*SE *(*B*)	*t*	*B*	*SE *(*B*)	*t*
Intercept (*b1*)	4.05	0.18	22.83***	3.79	0.21	17.49***
Block Number (*b2*)	− 0.14	< 0.01	− 17.6***	− 0.14	0.01	17.76***
Success Frequency (*b3*)	0.65	0.18	3.66***	1.94	0.63	3.07***
Success Frequency Squared (*b4*)				− 1.26	0.59	− 2.14*

^*^p < 0.05

^**^p < 0.01

^***^p < 0.001

The likelihood ratios test showed that the quadratic model (Fig. 5D) described the data better than the linear model (Fig. 5C) [*LR*(1) = 4.54, *p* = 0.03]. The quadratic model predicted peak motivation at a success frequency of 0.77.

## Discussion experiment 2

Experiment 2 showed that motivation depended on success frequency, and negatively on the block number. The relation between motivation and success frequency was best described by the quadratic model, showing the expected inverted-U relationship between motivation and success frequency with the apex at a frequency of 0.77. We did not make predictions on the success frequency that would result in peak motivation, but the estimated peak was at a higher frequency than the frequency of 0.5 that has been proposed by the theory of achievement motivation (Atkinson, 1957).

## General discussion

In two experiments, we tested the hypothesis that there is an inverted-U relationship between motivation and success frequency. In Experiment 1, the probability to continue increased linearly with the success frequency. The play duration, however, did show the expected inverted-U relationship with success frequency and the estimated peak motivation was at a frequency of 0.63. In Experiment 2, self-reported motivation showed the expected inverted-U relationship with success frequency and the estimated peak motivation was at a frequency of 0.77. In addition, motivation decreased with the block number.

Before interpreting these results, we will discuss study limitations. First, performance in the online game depended on stochastic factors such as the lifetime and the location of the virus. Successful performance required attentional effort of the participant to search the viruses and navigate the scene, but the task depended little on skill. Second, the average success frequency was quite low (0.6 in Experiment 1 and 0.54 in Experiment 2). These properties of the task might have influenced the relationship between motivation and success frequency. For instance, it has been found that the relation between the probability of success and motivation is stronger when individuals attribute success to effort (Kukla, 1972). Moreover, because high success frequencies were relatively rare, conclusions on the influence of high success frequencies on the motivation were based on relatively few data points.

We addressed motivation without dissociating between extrinsic motivation -the motivation to engage with a task to obtain rewards-, and intrinsic motivation -the motivation for the rewarding aspects of the task itself. We briefly speculate on the degree to which the motivation we studied was intrinsic or extrinsic. In Experiment 1, motivation was largely intrinsic as participants played for as long as they wanted without receiving reward. In Experiment 2, participants played for course credits and were thereby more extrinsically motivated. Our parameter of motivation, the QMI correlates well however with a standard assessment of intrinsic motivation: the intrinsic motivation inventory (IMI; Ryan, 1982).

The finding that motivation depends on success is consistent with the literature. Success signals competence and activities that make us feel competent are motivating (Ryan & Deci, 2017; White, 1959). A study among children showed that intrinsic motivation for a virtual reality game increased with success frequency (van der Kooij et al., 2018). Another study among adults showed that positive feedback increases motivation for an online game (Burgers et al., 2015). Also, providing adults feedback that they performed better than others increased their interest in a trivia game (Sansone, 1986). As two final examples, perceived physical competence of children is positively related with their physical activity (Lima et al., 2017) and self-efficacy, a concept related to perceived competence, predicts exercise behavior in adults (Neupert et al., 2009).

We specifically expected an inverted-U relationship where motivation would peak at an intermediate success frequency. We found tentative evidence of this relationship: the play duration and self-reported motivation showed the expected relationship although the probability to continue did not. One reason why the probability to continue did not show this relationship might be that in Experiment 1, quitting at a success frequency of 1 implied breaking a streak of ten successes which participant might have wanted to avoid, resulting in a high probability to continue in the highest frequency bin. The tentative evidence of an inverted-U relationship between motivation and success frequency is consistent with theories positing that intermediate challenge is most motivating (Atkinson, 1957; Csikszentmihalyi, 1990). Moreover, it shows the promise of quantifying motivation in terms of success frequency. Such a quantification facilitates application of motivation theories in adaptive game design, For instance, by informing the designer when the player should move to the next level of difficulty.

Although the idea of success frequency predicting motivation is promising, it must be noted that the relationship might be the other way around: motivation might enhance the success frequency by increasing performance (Nygard, 1975). For instance, it has been found that the opportunity to earn money increases accuracy in a reaching task (Gajda et al., 2016). Moreover, motivation enhances the velocity with which humans move (Sackaloo et al., 2015; Shadmehr et al., 2019). Finally, motivation might reduce the speed-accuracy trade-off as eye movements to more rewarding targets are not only faster but also more accurate (Manohar et al., 2015). The relationship between motivation and performance might thus be circular (Wulf & Lewthwaite, 2016). To uniquely show the influence of success on motivation, studies should manipulate success experiences independent of performance.

The generalizability of the results is constrained by the population. Recruiting students through the personal network of the students who ran the experiment and the University’s study participation system might have caused a baseline level of motivation for participating in the task. Moreover, in Experiment 2 mainly women participated. Yet, the results of Experiment 1, in which more men participated, were comparable. In addition, the personality of participants might have influenced the relationship between motivation and success frequency. Individuals with a strong motive to succeed prefer intermediate success probabilities whereas individuals with a strong motive to avoid failure prefer either high or low success frequencies (McClelland et al., 1953). The finding of peak motivation at a relatively high success frequency might thus be explained by the study population being largely driven by a motive to avoid failure.

An important avenue for future research is to assess how motivation depends on changes in success frequency. A constant balance between challenge and skill might not be optimally motivating (Baumann et al., 2016). It has been argued that the goal of a developing system is to keep exploring, rather than to settle into a stable state (Steels, 2004). A constant success frequency could signal a stable state and might therefore not be optimally motivating. Indeed, it has been shown that Flow is predicted by a deviation from expected success rather than by performance itself (Cowley et al., 2019). Ideally, studies on motivation as a function of changes in success frequency should measure motivation continuously while success frequencies change. Possibilities for measuring motivation on a more fine-grained timescale might be provided by measuring motivation with neurophysiological parameters such as cardiovascular measures (Keller et al., 2011), functional magnetic resonance imaging of areas in the dopaminergic system (Di Domenico & Ryan, 2017), or movement vigor (Summerside et al., 2018). Such fine-grained measures of motivation might also be able to determine on what timescale and how success experiences are integrated to form a motivational response.

To conclude, we found tentative evidence of an inverted-U relationship between motivation and success frequency. In addition, motivation decreased over time. This opens up a promising avenue of research on how motivation can be optimized by adapting difficulty to the success frequency.
